# Revitalizing local wisdom within character education through ethnopedagogy apporach: A case study on a preschool in Yogyakarta

**DOI:** 10.1016/j.heliyon.2024.e31370

**Published:** 2024-05-16

**Authors:** Syahria Anggita Sakti, Suwardi Endraswara, Arif Rohman

**Affiliations:** a Yogyakarta State University, Yogyakarta, Indonesia; b Universitas PGRI Yogyakarta, Indonesia

**Keywords:** Revitalization, Local wisdom, Character education, Ethnopedagogy, Kindergarten

## Abstract

Ethnopedagogy serves as an educational approach capable of reshaping societal dynamics by preserving cultural values, thereby reinforcing a multicultural national identity. This study explored the potential of ethnopedagogy in revitalizing local wisdom within early childhood character education in Indonesia. This case study was performed at Pertiwi Kindergarten in Puro Pakualaman, Yogyakarta, Indonesia. Data were gained through interviews and document analysis with teachers, parents, and school principals. Research data were checked for validity using data triangulation. The research findings demonstrated that ethnopedagogy enhanced children's awareness of local culture and nurtures character development by integrating local wisdom values into the learning process. Furthermore, involving local communities in character education improved community engagement in the educational journey, bolstering a sense of ownership over the school or learning environment, and strengthening social networks within the community. Ethnopedagogy is recommended to apply in early childhood education to develop children's character through the utilization of local wisdom.

## 1 Introduction

**Fig. 1:**
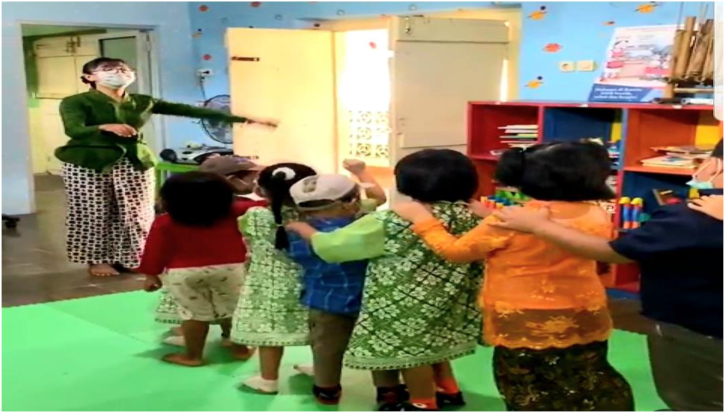
Traditional play “*Jamuran*” as a character building media among students.

**Fig. 2:**
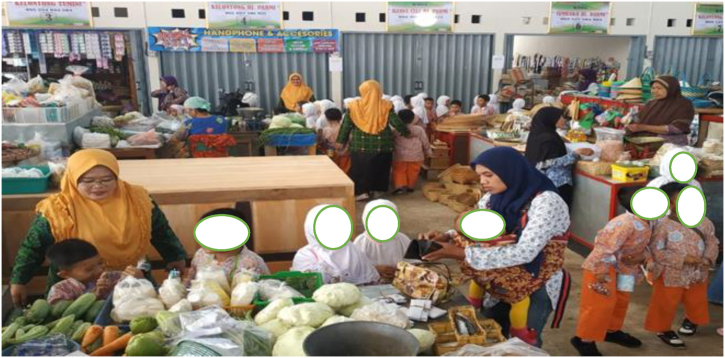
Students visiting traditional market to enhance local community enhancement in character education among students.

**Fig. 3:**
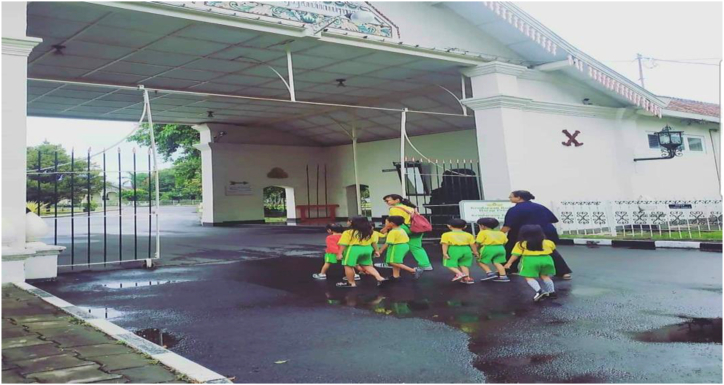
Students visiting Puro Pakualaman Yogyakarta to learn about local heritage.

**Fig. 4:**
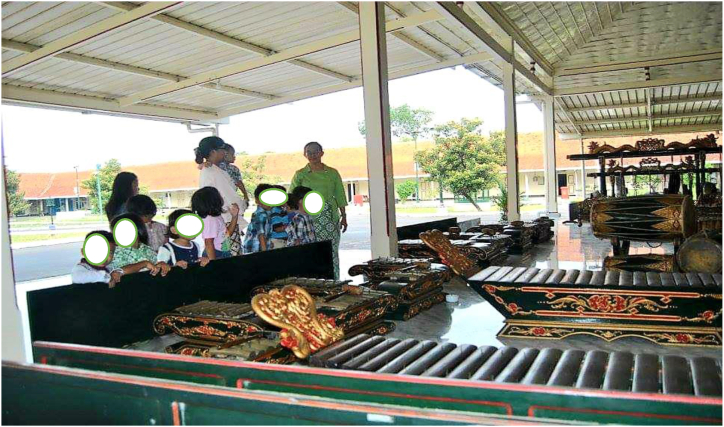
The Javanese cultural heritage Gamelan as a means of learning about Pakualaman.

**Fig. 5:**
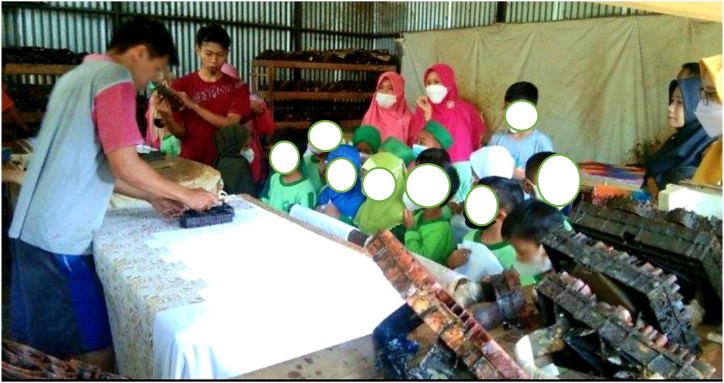
Visiting local batik artisans to engage local community in cultural heritage presentation.

Indonesia, a nation characterized by its rich tapestry of ethnicities, religions, and languages, embodies the spirit of unity in diversity, as encapsulated in its national motto, Bhinneka Tunggal Ika. Nevertheless, recent years have witnessed growing apprehension regarding the diminishing influence of local wisdom and values within the education system [1]. Recognizing the importance of infusing character education with indigenous wisdom, the Indonesian government has taken steps to rejuvenate these principles, particularly in early childhood education, a crucial period for shaping character and instilling fundamental values [2]. Within the realm of character education, the revitalization of local wisdom through an ethnopedagogical approach assumes paramount significance in fostering multicultural awareness and nurturing the internalization of indigenous values during early childhood [3]. Effective character education necessitates the inclusion of universally accepted values, such as honesty, tolerance, and cooperation, alongside the unique values inherent to local communities [4]. Through the ethnopedagogical approach, educators can seamlessly integrate local values and cultural elements into the learning process, enabling children to comprehend and appreciate the rich tapestry of cultural diversity and varied value systems. Furthermore, this approach contributes to the development of a positive cultural identity among children, strengthening their pride in their local heritage and values [5] (see Fig. 1, Fig. 2, Fig. 3, Fig. 4, Fig. 5).

Character education is fundamentally aimed at cultivating individuals who possess integrity, empathy, a sense of responsibility toward themselves and society, and a commitment to upholding truth and justice [6]. It plays a pivotal role in shaping a harmonious, cultured, and morally conscious society. The process of character education can take various forms, including direct instruction, emulation of role models, and experiential learning in everyday life [7]. Nurturing essential character traits from a young age is imperative, and one effective approach involves integrating local wisdom from the surrounding environment into education [8]. Local wisdom represents the cultural values inherited by a region, passed down through generations, and capable of molding an individual's foundational character [9]. Incorporating local wisdom into education not only strengthens cultural identity but also fosters robust character development among students [10]. Moreover, character education should align with local culture to ensure its relevance and applicability within the community while still preparing individuals for the global stage.

Character education for early childhood in Indonesia faces several pressing challenges, notably the inadequate understanding and integration of local wisdom into character development efforts, encompassing valuable values like cooperation, simplicity, unity, and environmental respect [11]. Another significant issue pertains to the quality of education, with many early childhood institutions emphasizing academics to the detriment of character education, resulting in a potential gap between the two [12]. Additionally, the pervasive influence of social media, as evidenced by the 73.7 million users in Indonesia, primarily among the youth, poses a contemporary challenge, potentially hindering physical activity and social engagement, which are vital for children's social and emotional development [13].

Character education in schools grapples with its own set of challenges. Despite attempts to incorporate it into the curriculum, many schools have not effectively implemented character education [14]. Recent data indicates that only approximately 40 % of schools in Indonesia have fully integrated character education, leveraging existing local wisdom [15]. Addressing the decline in character among children necessitates a collaborative, cross-sector approach involving families, schools, communities, and government. This collaborative effort should encompass the development of a comprehensive character education curriculum, training programs for teachers and parents in nurturing children's character, and the establishment of stricter oversight and regulations governing the influence of social media on early childhood [16].

Ethnopedagogy serves as a valuable approach for educators in effectively imparting the values and attitudes inherent to character education [17]. By delving into an understanding of local culture, teachers can tailor their teaching methods to align with the prevailing cultural and societal values [18]. Through the application of ethnopedagogical principles, character education can embrace cultural diversity, eradicating discrimination or prejudice against any particular cultural group [19]. This approach also ensures that character education remains pertinent to students' needs and aspirations, as it is rooted in the culture and traditions within which they mature. Consequently, the synergy between character education and ethnopedagogy becomes pivotal, guaranteeing its global utility while retaining relevance and applicability within local communities and cultures [20]. Ultimately, character education emerges as a potent tool for nurturing individuals with commendable values and attitudes while concurrently preserving and celebrating local cultural heritage.

The revitalization of character education via an ethnopedagogical approach unlocks the latent potential of cultural values that are often underutilized in shaping students' robust character development. Character education rooted in local wisdom involves a deliberate effort to rejuvenate local culture within the educational framework, contributing to the formation of a cohesive national identity [21]. In this context, Yogyakarta Special Region stands out as a province that has embraced culture-based character education. This commitment is exemplified by Regional Regulation no. 5 of 2015, which mandates the integration of culture-based character education into all aspects of schooling, spanning subjects, intracurricular activities, and extracurricular pursuits. Ethnopedagogy, as an approach, hinges on the application of local cultural values in the educational process, exemplifying its profound relevance in this educational context [22].

The ethnopedagogical approach plays a pivotal role in helping students comprehend the culture and traditions that surround them, fostering mutual respect among peers and nurturing a profound sense of pride in their local heritage [23]. Moreover, this approach serves as a catalyst for fortifying students' cultural identity, instilling essential character traits such as honesty, responsibility, tolerance, cooperation, and discipline [24]. By embracing ethnopedagogy, character education can help bridge cultural divides and eradicate any vestiges of discrimination or prejudice against specific cultures. This approach ensures that character education remains intimately connected to students' own needs and aspirations, as it is rooted in the culture and traditions that are intimately familiar to them. Consequently, revitalizing character education through an ethnopedagogical lens emerges as a potent strategy for nurturing individuals with robust character and a deep appreciation for cultural diversity, equipping them with the skills to thrive in diverse environments. According to Starostin and Olesov [25] ethnopedagogy can be used as a tool to support educational rights for indigenous peoples in various countries to shape the original character of the nation. The focus is on strengthening cultural identity and empowerment through an ethnopedagogical approach. Then similarly, Supriatna [26] discusses the use of ethnopedagogy in the context of human development and child education. This research highlights how educational practices embedded in cultural contexts can shape children's character development and understanding of values. This research is reinforced by Sayfiddinovich [27] who states that the ethnopedagogical approach is used to recognize and utilize the rich cultural knowledge possessed by students in the context of education.

Pertiwi Puro Pakualaman Kindergarten stands as an educational institution deeply entrenched in its cultural roots, where educational practices are profoundly influenced by cultural characteristics. Its unique curriculum deftly merges the national curriculum with the rich traditions of Javanese culture, as encapsulated in the Sestradi Puro Pakualaman Teachings. In this role, schools emerge as pivotal cultural hubs and agents of change, playing a vital part in nurturing and disseminating local wisdom and cultural values—essentially serving as social capital—in the dynamic landscape of Indonesian society [28]. The hallmark of Pertiwi Puro Pakualaman Yogyakarta school lies in its exceptional integration of culture throughout the learning process, meticulously woven into various learning centers within the institution. Here, character values are not just introduced but deeply understood, consistently upheld, and actively practiced in students' lives, both within the school premises and at home. The shared awareness among educators and stakeholders at Pertiwi Puro Pakualaman Kindergarten regarding the paramount significance of instilling character values is palpable in the intricately crafted curriculum and pedagogical approaches. The remarkable success achieved by Pertiwi Puro Pakualaman Yogyakarta Kindergarten in translating the noble cultural values of Puro Pakualaman into tangible outcomes underscores the profound importance of conducting an in-depth study to glean insights and inspiration.

In the context of early childhood education, this research examined the potential of employing an ethnopedagogical approach to seamlessly integrate local wisdom into kindergarten character education. The revitalization of local wisdom within character education provides compelling grounds for the application of ethnopedagogy in early childhood learning. Ethnopedagogy excels at forging robust connections between students and their cultural milieu, rendering learning more meaningful and pertinent [29]. As a means to enrich character education, ethnopedagogy empowers educators to identify and infuse character values embedded within local culture and traditions, thereby aiding students in comprehending and cherishing their cultural heritage [18]. Ethnopedagogy fosters a learning environment that is not only more relevant but also captivating for students, as it draws upon their own experiences, subsequently enhancing their engagement in the character development process [30]. Through this approach, students can gain a deeper understanding of cultural diversity and the character values that resonate across various cultures. In the course of this research, local wisdom containing character values suitable for incorporation into the kindergarten curriculum will be identified, and the effectiveness of the ethnopedagogical approach in introducing, imparting, and reinforcing these character values will be explored in depth.

## 2. Method

2

This research adopts a qualitative approach with a case study design to explore and comprehensively understand various systematic aspects related to the integration of local wisdom into character education in kindergarten settings [31]. Data collection encompasses a range of methods, including observation, interviews, and documentation. The case study methodology entails leveraging diverse data sources to provide a comprehensive exploration, description, and explanation of the subject under investigation, be it individuals, groups, programs, organizations, or events. This multifaceted approach necessitates the use of various data collection instruments, such as in-depth interviews, participant observation, documentation, recordings, and other physical evidence [32]. During the observational phase, researchers meticulously record and collect data in the form of field notes, capturing all pertinent events related to the integration of local culture into the learning process within the school environment. Simultaneously, secondary data, such as photographs and field notes directly generated by the researchers on-site, augment the primary data collection process. Subsequently, all gathered data undergoes a coding process, with careful attention to data categorization, culminating in data interpretation and ultimately, the presentation of findings [33].

The research involved school principals, teachers, and parents as subjects, encompassing key stakeholders in early childhood education. Qualitative analysis techniques were utilized to synthesize insights from interviews and observations. The case study method was chosen for its efficacy in examining the intricacies of revitalizing local wisdom in early childhood character education through an ethnopedagogical lens, focusing on a kindergarten setting. Yogyakarta, a cultural and educational hub in Indonesia, was selected as the research backdrop due to its rich cultural heritage and diverse social groups with varying perspectives on character and education. Pertiwi Puro Pakualaman Kindergarten, located in Yogyakarta, emerged as the natural research site, chosen purposefully to gain in-depth insights rather than seeking generalization, in line with the principles outlined by Lincoln & Guba [34].This selection was driven not only by the school's strong cultural characteristics but also its noteworthy success in effectively integrating character education with local wisdom, encompassing various cultural elements, such as art, music, dance, language, and customs, all integral to Javanese cultural identity.

### 2.1 Participants

The data sources for this study consisted of five teachers, one principal, and two parents of students, all selected using purposive sampling, a technique that focuses on choosing samples based on specific research criteria to ensure they possess the capacity to provide the required information regarding the revitalization of local wisdom in character education at Pertiwi Puro Pakualaman Kindergarten [35]. The informants were chosen based on the following criteria: 1) teachers who already have an educator certificate to teach in Early Childhood Education, 2) have attended character education workshops and have taught more than 5 years in Early Childhood Education with full enculturation, 3) demonstrate a strong understanding of school activities, and 4) have direct involvement by demonstrating a role in instilling character aligned with the Sestradi Teachings to children. In this study, purposive sampling also known as judgment sampling was used, where the sample was purposively selected based on its suitability to the research objectives [36].

### Ethical approval

This research was approved by the Institute for Research and Service of the Graduate School of Yogyakarta State University, Indonesia (1167/UN34.17/LT/2022) on August 22, 2022. Written consent was obtained from all participants.

### 2.2 Data collection

Data collection for this study encompassed observation, interviews, and documentation studies, all aimed at gathering pertinent and comprehensive data related to the research theme. First, through observation, researchers directly witnessed learning activities and teacher-child interactions in the kindergarten setting, enabling the collection of data on how local wisdom is integrated into early childhood character education through an ethnopedagogical approach. Observations were facilitated using tools such as cameras or field notes. Second, interviews with kindergarten teachers and parents provided valuable insights into their experiences with early childhood character education and the application of local wisdom in the learning process. These interviews were conducted either face-to-face or via telephone. Third, documentation, comprising video recordings, photographs, and written materials like curriculum documents, learning programs, and student progress notes, offered information on how local wisdom was put into practice in learning activities and student development, both before and after implementing the ethnopedagogical approach. Guided interviews were the chosen method, focusing on specific interview subjects or areas but allowing for flexibility to adapt as new insights emerged during the interviews, and additional topics were explored beyond the core questions posed to the respondents [37].

### 2.3 Data analysis

The qualitative data analysis approach employed in this research adhered to an interactive model encompassing three key stages: data reduction, data presentation, and drawing conclusions [36]. Following the collection and processing of qualitative data, the subsequent phase involved data analysis, which could encompass descriptive analysis methods, interpretive analysis, and comparative analysis. The overarching goal of data analysis was to derive insights and conclusions pertaining to the research theme. In the case of this study, focused on the revitalization of local wisdom in early childhood character education through ethnopedagogy in Yogyakarta Kindergartens, data analysis unfolded across three distinct stages: pre-analysis, data analysis, and data interpretation. The collected data underwent an in-depth qualitative descriptive analysis, entailing the meticulous process of data collection, reduction, presentation, and ultimately, drawing meaningful conclusions from the acquired data.

Data reduction involved selecting, focusing, simplifying, grouping, and transforming raw data from field reports throughout the research, with this process continuing during data collection through activities like summarizing, coding, and note-taking. In this study, data reduction primarily entailed grouping data based on research subjects and eliminating irrelevant information. The data display phase systematically presented information in the form of narrative text, graphics, or diagrams to facilitate easy comprehension and draw conclusions, with the presentation being limited by the requirement that it should be organized in a way that could lead to meaningful conclusions. Data was presented using various methods, including images, tables, and narratives, with the goal of consolidating information. Drawing conclusions was a comprehensive process that sought to derive meaning from the collected data by identifying similarities and differences to address existing problems, and these conclusions underwent verification throughout the research process to ensure data accuracy and accountability. After the data had been displayed and described, the study obtained answers to its research questions, allowing for the formulation of general conclusions and the generalization of research findings.

### 2.4 Data validity

Triangulation, a research technique, was employed to enhance data accuracy and validity by collecting information from multiple sources and utilizing various data collection methods. This approach aimed to mitigate bias and bolster confidence in the research outcomes. The process of triangulation involved gathering data from diverse sources to cross-verify the obtained results [38]. In this particular study, researchers acquired data through observation, interviews, and documentation, employing triangulation to heighten data validity and foster trust in the research findings. During the data collection phase, the research team ensured data validity by validating the interview instrument on three fronts: content, construct, and empirical validation. Content validation involved seeking guidance from experts in Javanese Culture, faculty lecturers from Gajah Mada University. Construct assessment was conducted by a lecturer from the arts and language faculty at Yogyakarta State University, while character education experts, who were lecturers at Yogyakarta State University's Postgraduate School, were selected to evaluate empirical validation.

## 3 Results

### 3.1 Ethnopedagogy within character education in preschool

Culture plays a pivotal role in shaping a child's character, especially during early childhood when children develop their character through daily experiences within their cultural milieu. Effective character education must be sensitive to the cultural context and engage local communities [39]. The development of an ethnopedagogical framework seeks to establish a harmonious relationship between educators and students, fostering the acquisition of knowledge, skills, and cultural understanding to achieve educational objectives. In pursuit of these educational goals, educators are expected to possess a minimum of three fundamental teaching competencies: (1) an understanding of students and their learning and developmental processes within specific social and cultural contexts; (2) proficiency in curriculum content and objectives, along with effective teaching methods; and (3) expertise in teaching that considers both content and students, encompassing the creation of productive classroom environments and the implementation of assessments [40]. In essence, ethnopedagogy evolves from educators' comprehension of their own cultural values, as well as those of their students, in the management of the teaching and learning process. As one informant explained regarding the ethnopedagogical approach in the learning process:

“*The application of this method can help children understand local values better. We came to understand that there are many cultural values in the Puro Pakulaman environment that can be explored and used as learning resources for students. This can strengthen children's character and prepare them to face the future. There are many local values that we take advantage of. For example, the values of mutual cooperation, cleanliness and courtesy. "We teach these values through various activities such as playing, singing and telling stories in learning centers at school*" (informant 1)

The insight shared by one of the informants underscores that kindergarten can serve as a highly effective setting for nurturing positive values and molding children's character. Through activities tailored to their developmental stages and learning requirements—such as games, storytelling, songs, and traditional games—children can readily integrate values like honesty, cooperation, and mutual respect. Leveraging the cultural values inherent in the school environment, children not only learn about them but also acquire the skills to apply these values in their everyday lives, thereby fortifying their character for the challenges that lie ahead in their future.

Various cultural values, including mutual cooperation, cleanliness, and good manners, can be effectively imparted to children through enjoyable activities like play, singing, and storytelling. Such engaging methods enable children to grasp and internalize these values more readily. Teaching these local values holds significant importance in molding children's character and fostering their growth as better individuals. Moreover, incorporating traditional cultural values into the character development process helps children establish a deeper connection with their cultural heritage. This, in turn, reinforces their sense of identity and instills a profound pride in their cultural roots. This aligns with insights shared by one of the informants, who highlighted this approach as follows.

" *At the learning centers, students have the opportunity to engage in various activities. They can immerse themselves in learning the art of batik, a quintessential aspect of Yogyakarta's culture. Additionally, they can partake in traditional dance sessions and practice Javanese songs that convey moral messages and virtues, serving as a vehicle for character development. (*informant 2*)*

As described by informant 2, this approach exemplifies the teacher's application of ethnopedagogy within the school's curriculum, seamlessly integrating it with the Pakualaman cultural curriculum, which has been an enduring heritage since ancient times, serving as a valuable tool for educating the younger generation of Pakualaman. Teachers have significant roles in developing children's character in kindergarten. Teachers need to have a deep understanding of local cultural values and should be attentive to variations in children's experiences and needs. Additionally, teachers must serve as exemplary role models in their behavior and actions. A comprehensive and integrated approach to character education proves highly effective in kindergarten settings. This approach encompasses the development of children's cognitive, emotional, and behavioral dimensions while actively involving parents and the local community in supporting character formation [41]. In essence, ethnopedagogical research in character education within kindergarten underscores the profound impact of cultural influences on character development. Therefore, effective character education must consider the cultural context and engage all stakeholders within the kindergarten environment to successfully cultivate positive character traits. Insights from schools further validate this notion, demonstrating that activities emphasizing cooperation, tolerance, and empathy play a pivotal role in helping children recognize the significance of these positive values in their lives, aligning with the perspective of the informant.

“Teaching good habits such as discipline, honesty and responsibility can be done by involving existing local communities. Good habits like these can help children build strong and positive characters. By visiting craftsmen's centers and traditional markets, students can help instill positive character in children and help them become independent, responsible individuals and have strong positive values. (informant 3)

“ Fostering virtues like discipline, honesty, and responsibility can be effectively accomplished through collaboration with the local community. These commendable habits play a crucial role in nurturing resilient and upright character traits in children. Activities such as visits to artisan workshops and traditional markets enable students to internalize these positive character traits, paving the way for them to become self-reliant, responsible individuals grounded in strong moral values (informant 4)

Engaging local communities in the learning process can provide children with a tangible and enduring understanding of positive values like discipline, honesty, and responsibility. A practical approach to achieving this is by arranging visits to artisan centers and traditional markets, which offer students insights into the local way of life and culture. These visits create opportunities for students to directly interact with craftsmen and traders, witnessing firsthand their disciplined, honest, and responsible work ethics. Furthermore, students can learn the art of maintaining favorable customer relationships and preserving traditional values in their business activities. Through these experiences, students come to appreciate the paramount importance of discipline, honesty, and responsibility in both daily life and business endeavors. Consequently, these encounters aid in the development of strong and positive character traits, fostering independence and responsibility while instilling robust positive values. Leveraging local resources like artisan centers and traditional markets not only imparts knowledge about local cultural values but also allows local communities to play a pivotal role in teaching positive values and cultivating the strong and positive character of students.

Values instilled through Ethnopedagogy activities along with observational information on how teachers instill these values.

**Table:** 

No	Ethnopedagogy Activities	Values Embraced	Observed Teacher Actions
1.	Local Traditional Stories	Cooperation and solidarity values highlighted in shared stories, respect for traditional moral values	The teacher enthusiastically selects stories that contain positive values. Invites students to discuss the moral message that can be taken from the story.
2.	Using Local Language in Learning	Respect for language diversity. Effective communication in the local context. Understanding of the importance of preserving local languages	Teachers integrate local vocabulary in daily teaching. Encourage students to communicate in local languages, enhancing their understanding of cultural identity.
3.	Visits to Local Historic Places	A sense of love and responsibility for local history. Connection between past, present and future. Respect for ancestors	The teacher guides students to recognize and understand local historical places. Students explore and present the results of the visit to the local cultural heritage.
4.	Collaboration with Local Community	Involvement in social activities. Cooperation and leadership skills. Appreciation of the role of the individual in society	Teachers organize collaborative projects with the local community. Students engage in charity or social projects, strengthening their involvement in the local community.

Effective communication with the teacher in charge of the learning center holds great significance to ensure that children derive maximum benefit from their experiences there. Teachers play a pivotal role in this regard by implementing child-centered learning methods and fostering direct interactions between themselves and the children. Furthermore, teachers diligently monitor the individual development of each child, offering recognition and praise as incentives to motivate them to persist in their learning and accomplishments.

"*The children display immense enthusiasm when engaging in activities at the arts and culture center. Within this learning environment, we create a delightful atmosphere that allows us to instill fundamental character traits in our students. As our school is situated in the Puro Pakualaman area, we benefit from a wealth of learning ecosystems rooted in our rich cultural heritage*." (informant 5)

From this interview, it becomes evident that the cultural ecosystem within schools holds the potential to effectively nurture children's character, provided it is managed adeptly. It is essential to employ appropriate pedagogical methods and ensure ample direct interaction between educators and students to maximize the benefits for each child. Children exhibit higher levels of enthusiasm and engagement in learning when they can actively participate in hands-on, interactive experiences like those described. Art centers, for example, offer a platform for children to explore diverse avenues of creative self-expression, fostering both individuality and collaboration as they work collectively to produce beautiful and meaningful artworks. Simultaneously, cultural centers afford children the opportunity to immerse themselves in various character portrayals drawn from traditional stories, aiding in the development of empathy and an understanding of differing perspectives. Additionally, these centers facilitate enhanced communication skills and the cultivation of crucial social competencies for children's everyday lives. Consequently, arts and culture centers emerge as a delightful and enriching educational experience for children, imparting essential qualities such as cooperation, creativity, empathy, and social adeptness.

Based on these findings, the ethnopedagogical approach is grounded in the acknowledgment of culture's significant influence on education. Consequently, the components utilized within the ethnopedagogy-based character education model encompass the family, school, and community. The character attributes attainable through ethnopedagogy-based character education include.

**Table:** 

No	Education Unit	Target Characteristics
1.	Family	Pride in culture and self-identity, empathy and tolerance, cooperation and togetherness, discipline and responsibility.
2.	School	Pride in culture and self-identity, empathy and tolerance, cooperation and togetherness, discipline and responsibility.
3.	Community	Pride in culture and self-identity, empathy and tolerance, cooperation and togetherness, discipline and responsibility, honesty and integrity

#### 3.1.1 Integrating local culture into learning activities

Incorporating local culture into kindergarten education represents a vital initiative aimed at nurturing children's cultural identity and awareness right from their formative years. Research findings underscore the significance of infusing local culture into kindergarten learning, as it serves to familiarize children with the rich tapestry of cultural diversity within their surroundings [20]. This approach contributes significantly to fostering tolerance and mutual respect among children from different cultural backgrounds. An informant eloquently expressed this idea by stating,

"*In my role, I continually strive to discover creative ways for acquainting children with our local culture. This involves imparting regional songs, teaching traditional dances, and sharing folklore that encapsulates our heritage. Moreover, I actively engage students' parents, inviting them to partake in activities steeped in our local cultural themes. I firmly believe that it is paramount for our students to develop a deep-seated familiarity and affection for our local culture starting at a tender age. This early exposure not only fosters a profound appreciation for our cultural heritage but also instills a sense of responsibility for its preservation and continuation into the future*." (Informant 6)

As part of the journey to integrate local culture, children are encouraged to acknowledge the cultural values passed down by their forebears. The early exposure and incorporation of local culture into their lives serve as a potent means to fortify their sense of local identity [3]. The integration of local culture into their learning experience can serve as a wellspring of inspiration for children, sparking their creativity and innovation. They can apply the distinct aspects of local culture they have learned in various creative outlets, such as art, dance, music, and more. This not only enhances their creativity but also fosters a sense of stewardship for the environment, a heritage entrusted to them by their ancestors.

By infusing these values into the kindergarten learning experience, teachers can instill in children a profound sense of environmental stewardship from their earliest years. The integration of local culture into their educational journey further serves as a potent tool for reinforcing social values, including mutual cooperation, solidarity, and a strong sense of togetherness. This holistic approach plays a pivotal role in molding children's characters, nurturing them to become socially adept and independent individuals. As succinctly articulated during an interview with the school principal:

*"In essence, the integration of local culture into kindergarten learning yields a host of positive outcomes, including the development of diversity, identity, creativity, environmental awareness, and crucial social values in our children. As a result, the promotion and further enhancement of this integration within both kindergarten and higher levels of education remain imperative. Collaboration with parents to infuse local culture into our educational programs is a priority. We advocate for the seamless integration of local culture as an integral component of the curriculum, and we enthusiastically endorse the organization of activities steeped in local cultural themes. This commitment ensures that our children not only inherit their cultural legacy but also contribute to its vibrant continuity."* (Informant 1)

Integrating local culture into education extends beyond mere familiarity with one's own heritage; it encompasses the appreciation of cultural diversity on a global scale. Children, through this approach, gain an understanding that the world comprises a rich tapestry of cultures, and they learn to embrace and respect these differences. The introduction of local culture from an early age serves as a pivotal tool for children to forge a stronger connection with their own cultural identity, fostering a sense of pride and appreciation for their cultural heritage. This exposure not only nurtures a deeper understanding of one's roots but also equips children with valuable skills for engaging with individuals from diverse cultural backgrounds [42]. They develop essential social competencies, including tolerance, empathy, and an innate understanding of others. In the pursuit of integrating local culture into kindergarten learning, it becomes imperative to craft a fitting curriculum and employ experiential learning methodologies. Collaboration between educators, school administrators, and local communities becomes pivotal to gain insights and access the requisite resources. In summation, integrating local culture offers children a profound and meaningful educational experience from an early age, nurturing their connection to their own culture and preparing them to appreciate the rich diversity of the broader world.

### 3.2 Engaging local community into the character education

Research conducted in kindergartens underscores the paramount significance of engaging local communities as a potent avenue for character education implementation. This is predicated on the notion that involving local communities in early childhood education bolsters the character values being imparted within school settings. By actively enlisting parents and the broader community in the early childhood education process, we can fortify the character values being instilled in schools. Parents and the surrounding community serve as tangible role models, exemplifying the daily application of virtues such as respect for diversity, honesty, cooperation, and responsibility, among others. This synergy with the local community enables children to glean insights about their immediate environment and their role in safeguarding it, thus fostering an enhanced understanding of environmental values and sustainability [43]. Importantly, the school ensures that the values conveyed by the local community align with those they seek to instill in children. Collectively, these research findings underscore the pivotal role of community involvement in the implementation of character education. Through the collaborative efforts of schools and local communities, children can be guided to emerge as individuals imbued with robust character values, poised to confront future challenges. This resonates with the sentiment expressed by one informant:

*With the involvement of parents and neighborhood residents in character education activities in our kindergarten, we are witnessing real and positive changes. Children show high acceptance of the character concepts imparted, exhibiting increased enthusiasm and motivation to develop good attitudes and behaviors.* (Informant 7)

The involvement of local communities is undeniably pivotal in the successful implementation of character education within kindergartens. By engaging parents, local residents, and community leaders, children are afforded the invaluable opportunity to glean wisdom from the examples and experiences of the adults in their immediate environment. The integration of local communities, including traditional markets and local artisans, into the realm of children's character education represents a concerted effort to enrich their character development through non-formal education channels. This community engagement facilitates children's comprehension of local values, fosters empathy and environmental stewardship, and cultivates mutual respect and cooperation within society. As aptly articulated by one informant:

*"Our school actively collaborates with the traditional markets and local craftsmen in our vicinity. We extend invitations to them to participate in various school activities, including competitions centered around handicrafts and batik making. In addition to these events, we organize field trips to these traditional markets and local artisan workshops, providing children with direct, hands-on exposure to essential values like diligence, creativity, and the importance of working together as a community."* (Informant 1)

**Table:** 

No	Theme	Target Characteristics
1.	Visiting traditional market	Appreciating and caring for the social environment
2.	Visiting local artisans	Creativity and Collaboration
3.	Attending Batik workshop	Creativity and Independence
4.	Attending traditional ceremonies	Togetherness and Mutual Respect

*“ Children not only gain a deeper understanding of the positive values inherent in their immediate community, but also develop an appreciation for the cultural diversity that exists around them.* (Informant 8)

These findings underscore the significant benefits of involving local communities in enhancing children's learning experiences through contextually relevant themes and activities tied to local and traditional values. Consequently, the pivotal roles of parents and educators become evident in facilitating children's participation in these non-formal character education initiatives. Through such engagement, children not only acquire a deeper understanding of the positive values inherent in their immediate community but also develop an appreciation for the cultural diversity that thrives within their surroundings, thereby fostering tolerance and respect for differences. Moreover, the involvement of local communities serves to strengthen children's connection to their environment, nurturing a heightened sense of care and responsibility towards it [44]. The collaborative efforts between schools and local communities are purposefully directed toward cultivating an educational environment that places the cultivation of strong character traits at the forefront. This represents a positive stride toward nurturing a more ethically minded and responsible generation.

## 4 Discussions

Understanding local wisdom in imparting character education to students holds profound significance as it empowers them to cultivate strong character, fostering their growth as exemplary citizens who are deeply attuned to their cultural surroundings. This approach nurtures cultural awareness and underscores the imperative of preserving their cultural heritage and native language, facilitating a mindset that is open and tolerant toward differences. Grasping local wisdom equips children with the capacity to develop empathy and mutual respect for the cultural and value systems of others. It ingrains the notion that diverse perspectives and values are unique and invaluable facets of people's lives [45]. Moreover, by comprehending local wisdom, children assimilate essential character values such as honesty, diligence, cooperation, empathy, and mutual respect [46]. This not only encourages them to respect differing cultures and values but also primes them to be receptive to learning and adapting to a broader, more diverse environment. Hence, understanding local wisdom stands as a pivotal cornerstone in the character development of children.

Albert Bandura's theory [47] posits that individuals acquire knowledge and skills primarily through observation and interaction with the individuals in their immediate social milieu. In the realm of character education, the ethnopedagogical approach, which involves local communities, provides children with invaluable opportunities to observe and engage with community members who embody the local cultural values and norms. Consequently, children can grasp and internalize the positive attributes embedded within their culture, such as tolerance, cooperation, and respect for differences. The process of learning and the formation of one's character are inherently intertwined with social interactions and the environment. In the ethnopedagogical approach, local culture assumes a pivotal role as a fundamental element within children's social environment. Through these interactions with local culture, children actively construct their comprehension of the values, norms, and ethical standards prevalent in their society [48]. Thus, the ethnopedagogical approach, which seamlessly integrates local culture into education, serves as a catalyst for the cultivation of robust character and a deeper understanding of social values.

Revitalizing local culture within the framework of character education during early childhood is a crucial endeavor. Nurturing good character in children serves as a cornerstone for their future as upstanding individuals. Employing an ethnopedagogical approach to rejuvenate local wisdom in early childhood character education has the primary goal of acquainting, cultivating, and perpetuating these cultural treasures within society [49]. This initiative holds significant importance, given that many local traditions face the risk of fading into obscurity due to a lack of attention and appreciation from the broader community. By embracing an ethnopedagogical approach, children are encouraged to engage with local wisdom in a joyful and continuous manner throughout their daily lives [50]. This research underscores that the revitalization of local wisdom through ethnopedagogical methods in early childhood character education not only facilitates children's understanding and appreciation of the cultural heritage around them but also fosters attitudes of tolerance and mutual respect for cultural diversity. Moreover, it fortifies their sense of cultural and national identity and bolsters their self-confidence, enabling them to play an active role in preserving and nurturing local wisdom within their communities.

Recent trends show an increasing awareness of the need for character building at an early age. Character education is not only considered as a complement to the curriculum but as an integral part of early childhood learning. The education curriculum in Indonesia is increasingly shifting to project-based learning. This is in line with efforts to foster the six Pancasila student profiles, which involve aspects of character, personality and skills [51]. Ethnopedagogy naturally supports a culture-based learning approach. In the Indonesian context, where cultural diversity is rich, ethnopedagogy can be an effective tool for integrating local values into early childhood learning. Project-based curriculum emphasizes hands-on experience and practice. Ethnopedagogy with the use of folklore, traditional games and visits to historical places can synergistically support this approach in the Indonesian context. The research [52] states that ethnopedagogy provides a space for students to recognize character built from local culture and can provide suggestions for best practices that can be adopted and adapted to the context of early childhood education in Indonesia. This can be a valuable guide for the application of ethnopedagogy in local contexts. By integrating this research with trends in character education, ethnopedagogy can strengthen the rationale of the research. This is crucial to ensure the relevance and urgency of the research in addressing the challenges of early childhood character education in Indonesia, as well as making a positive contribution to the development of learners in accordance with the values of Pancasila. This is important to convince the relevance and urgency of the research in facing the challenges of early childhood character education in Indonesia, while making a positive contribution to the development of students in accordance with the values of Pancasila.

The importance of ethnopedagogy is also very relevant for early childhood students, especially in kindergarten. At this age, children are very open to environmental influences and have a high receptivity to the cultural values around them. Therefore, applying ethnopedagogy can help create a learning environment that suits the needs and cultural context of young children. For example, in a kindergarten context, an ethnopedagogical approach can integrate local stories, traditional games and other creative activities that reference local cultural heritage. Thus, children can develop a deeper understanding of their cultural values from an early age. In comparing the findings of the ethnopedagogy research with previous research on cultivating cultural values in early childhood, the authors can highlight the differences in approaches and outcomes achieved. Ethics, social norms and cultural values may be more easily internalized by children through an ethnopedagogical approach that utilizes their cultural context.

The revitalization process is significantly enhanced through the integration of local culture into learning. This integration not only boosts the effectiveness of learning but also ensures that students acquire subject matter knowledge in a context that resonates with their daily lives. The inclusion of local culture in education can be achieved by immersing students in direct experiences with their local culture, including visits to significant cultural sites and active participation in cultural activities like cooking local cuisine, traditional dancing, and crafting indigenous handicrafts [53]. Additionally, enriching learning materials with local resources, such as utilizing local sources, incorporating books or articles pertaining to local history, and integrating local language and terminology into teaching (both in everyday language and specific subject matter) can further facilitate this integration. Moreover, forging connections with local communities and cultural organizations proves instrumental in supporting the learning process and providing essential support for various learning activities [49].

Ethnopedagogy can stimulate positive changes in students' attitudes towards learning, as they feel recognized and valued in their cultural context. This finding can provide a context for the results of the study that showed positive changes in students' attitudes towards learning after the application of ethnopedagogical methods [54]. Other studies state that ethnopedagogical methods can help in the development of students' social skills as they learn in a more inclusive and community-oriented context. Therefore, the results of the study showing changes in students' social skills may be in line with the findings of previous studies [55]. The application of ethnopedagogy can also contribute to students' positive identity building. This happens because students see how their culture is valued and integrated in the learning environment [56]. This research explores ethnopedagogy with a more holistic approach, involving not only curriculum aspects but also the active involvement of local communities in the early childhood learning process. This research specifically emphasizes local relevance in the context of ethnopedagogy, presenting strong evidence that this approach can effectively foster local character in early childhood in kindergarten. Previous research may have focused more on the introduction of cultural values without considering the extent to which learning materials can be linked to children's everyday cultural context. This research highlights the importance of contextualized and relevant learning materials.

The integration of local culture into the learning process should be comprehensive, extending beyond specific subjects to permeate the overall learning environment and interactions between teachers and students [57]. This integration entails carefully selecting educational content that aligns with local culture, organizing activities that reflect local traditions, and leveraging the immediate surroundings as valuable learning resources. By infusing local culture into education, students are more likely to develop a heightened sense of pride and affection for their heritage, ultimately bolstering their national identity. This approach also fuels students' motivation to learn, as it encompasses a diverse array of learning dimensions through various engaging activities. As students delve into their local culture, they gain a deeper understanding of the distinctiveness and beauty of their regional heritage, instilling in them a profound sense of pride in their cultural identity and fostering a stronger sense of belonging and appreciation for their culture. Furthermore, by acquainting students with local culture, they are better equipped to comprehend the norms and values cherished by their local community, which in turn cultivates attitudes of tolerance and mutual respect between cultures. The incorporation of local knowledge within education not only facilitates the development of strong character but also fosters a deeper understanding of societal values.

Ethnopedagogy allows the use of learning materials that are directly related to local culture. This helps students to better understand and internalize local values. With a focus on local culture, ethnopedagogy helps in the development of students' social skills, as they learn to interact within their own cultural context. Early childhood is in a phase of identity development. Ethnopedagogy helps them identify themselves within their cultural context, building a sense of pride in local heritage [58]. Early learners understand abstract concepts more easily through direct experience. Ethnopedagogy provides real experiences that are relevant to their cultural context. Strong character and values instilled at an early age tend to form a solid foundation for later development [59]. Ethnopedagogy tends to use active learning methods, such as local folklore, traditional games, and visits to historical places. These provide hands-on experiences that may be lacking in previous research. Involving the community, using culturally relevant learning materials and focusing on children's identity development make ethnopedagogy a strong option in the context of early childhood education. Comparisons with previous research show significant progress in this approach, illustrating success in achieving character and cultural values education goals.

### 4.1 Implications

The research findings unequivocally demonstrate that incorporating local culture into the learning process can significantly enhance students' comprehension of educational content. This integration of culture into various subjects not only renders the course material more pertinent but also facilitates a deeper and more enthusiastic engagement from students. Furthermore, the inclusion of local culture in the curriculum equips students with practical applications for the acquired knowledge in their day-to-day lives, thereby making their learning experiences more practical and personally relevant.

### 4.2 Limitations

This research adopted a case study approach within a single kindergarten located in Yogyakarta. Consequently, it is essential to recognize that the outcomes may not be readily generalizable to a broader population. While the study does shed light on the significance of applying ethnopedagogical methods to character education in Indonesia, it is crucial to acknowledge that the findings primarily pertain to the specific context of this particular case. Furthermore, the research encountered challenges in gathering secondary data, including historical and cultural information specific to the Yogyakarta region, which served as the study's focal point.

Despite these inherent limitations, it is worth emphasizing that this study holds substantial potential for contributing to the advancement of character education in Indonesia. Notably, it introduces the ethnopedagogical approach as a valuable alternative for developing and integrating local wisdom values into character education initiatives within the Indonesian context.

## 5 Conclusions

The research findings indicate that revitalizing local wisdom through ethnopedagogy can effectively stimulate children's active engagement in character education while preserving their local cultural heritage. This approach fosters an understanding and appreciation of cultural diversity, cultivating tolerance and open-mindedness towards differences. The pivotal role of the teacher as a facilitator in conveying the essence of local wisdom and its practical application in daily life cannot be overstated. Effective character education transcends moral values, necessitating the inclusion of local culture and active participation of local communities. The integration of local culture and community involvement within character education programs holds the potential to shape a generation with strong character and a profound ability to comprehend and value cultural diversity. Consequently, it is strongly recommended to incorporate ethnopedagogy into early childhood education as a means of nurturing children's character development through the rich tapestry of local wisdom.

## Funding statement

This research received no specific grants from funding agencies in the public, commercial, or nonprofit sectors.

## Data availability statement

The data used is confidential.

## Additional information

No additional information is available for this paper.

## CRediT authorship contribution statement

**Syahria Anggita Sakti:** Writing – original draft, Resources, Conceptualization. **Suwardi Endraswara:** Supervision. **Arif Rohman:** Supervision, Methodology.

## Declaration of competing interest

The authors declare that they have no known competing financial interests or personal relationships that could have appeared to influence the work reported in this paper.
